# Extracellular Vesicles and Tumor-Immune Escape: Biological Functions and Clinical Perspectives

**DOI:** 10.3390/ijms21072286

**Published:** 2020-03-26

**Authors:** Stefania Raimondo, Marzia Pucci, Riccardo Alessandro, Simona Fontana

**Affiliations:** 1 Department of Biomedicine, Neurosciences and Advanced Diagnostics (Bi.N.D), Section of Biology and Genetics, University of Palermo, 90133 Palermo, Italy; stefania.raimondo@unipa.it (S.R.); marzia.pucci@unipa.it (M.P.); riccardo.alessandro@unipa.it (R.A.); 2 Institute of Biomedicine and Molecular Immunology “A. Monroy”, National Research Council, 90146 Palermo, Italy

**Keywords:** extracellular vesicles (EVs), cancer immune tolerance, immune checkpoints, PD-1/PD-L1 axis

## Abstract

The modulation of the immune system is one of the hallmarks of cancer. It is now widely described that cancer cells are able to evade the immune response and thus establish immune tolerance. The exploration of the mechanisms underlying this ability of cancer cells has always attracted the scientific community and is the basis for the development of new promising cancer therapies. Recent evidence has highlighted how extracellular vesicles (EVs) represent a mechanism by which cancer cells promote immune escape by inducing phenotypic changes on different immune cell populations. In this review, we will discuss the recent findings on the role of tumor-derived extracellular vesicles (TEVs) in regulating immune checkpoints, focusing on the PD-L1/PD-1 axis.

## 1. Introduction

Tumors adopt numerous strategies to manipulate the surrounding microenvironment to guarantee and support their development. One of the more powerful strategies through which cancer cells protect their growth concerns the possibility to evade the immune system. Within tumor microenvironment (TME) several mechanisms have been described to be responsible for immune tolerance, ultimately promoting tumor proliferation and metastasis. Cancer cells can induce immune cell death via the FasL/Fas and PD-L1/PD-1 pathways, resulting in a decrease in the number of T-cells and NK cells. In addition, they also recruit the immuno-suppressive Regulatory T cells (Tregs) and myeloid-derived suppressor cells (MDSCs) that inhibit CD8+ T-cells, resulting in tumor immune escape.

To deeply investigate how cancer cells can activate these immune escape mechanisms, in recent years researchers have focused on the study of extracellular vesicles (EVs), a heterogeneous group of lipoproteic structures, released from all cell types [1,2]. It has now been widely demonstrated that EVs derived from tumor cells (TEVs) can promote tumor-mediated immune suppression creating a tumor-friendly microenvironment [3,4]. Many studies are specifically focused on small extracellular vesicles (sEVs), to date also named exosomes, a well-characterized subtype of EVs playing a pleiotropic role in different key processes of tumor formation and progression; in fact, EVs are involved in tumor microenvironment (TME) remodeling as angiogenesis [5,6,7], invasion [8,9], metastasis [10,11,12], and resistance to therapies [13,14]. sEVsare nano-sized (40–100 nm) membrane-delimited vesicles that are secreted by almost all cell types under both normal and pathological conditions. They are usually detected in biological fluids like blood, urine, ascitic fluid and others. sEVs transport various biomolecules, such as proteins, messenger RNAs (mRNAs), microRNAs (miRNAs), and long non-coding RNAs (lncRNAs) [2,3]; common exosomal markers include HSp70, CD9, CD63, and CD81 [4,5]. The release of sEVs is a complex process that the cells execute following multiple steps in which different proteins are involved. Among those, neutral sphingomyelinase 2 (nSMase2) [15,16], phosphorylated synaptosome-associated protein 23 (SNAP23) [17,18] and Ras-related RAB proteins (RAB27A/RAB27B) [19,20,21] regulate sEV secretion from different cancer cells like breast cancer [15,16], hepatocellular carcinoma (HCC) [17,18], and colorectal cancer [17,20].

In the next sections, we will discuss the scientific evidence showing that EVs and in particular sEVs released by cancer cells play a key role in promoting the immune escape of the tumor, specifically modulating the behavior of each cellular component of tumor immune microenvironment. Particular emphasis will be given to the role that tumor-derived extracellular vesicles (TEVs) have in regulating immune checkpoint directly activating the PD-L1/PD-1 axis.

## 2. Mechanisms of the TEVs-Mediated Immunoescape

**Figure 1:**
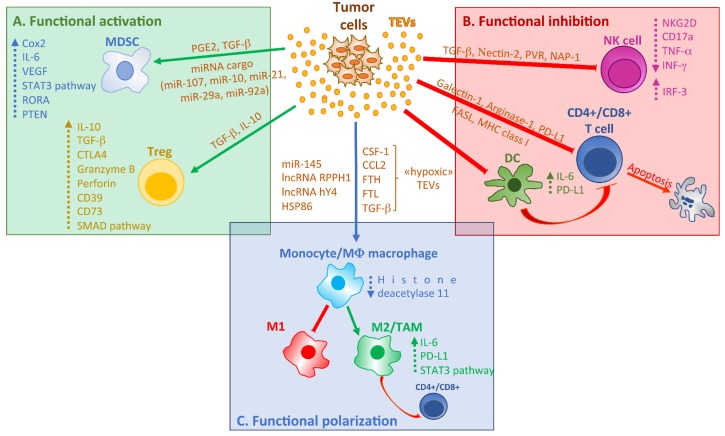
Overview of TEV-mediated mechanisms modulating the immune response in the tumor microenvironment. TEVs can help tumor cells to escape from the immune system by adopting several strategies such as the functional activation of cells having an immunosuppressive activity (**A**), the functional inhibition of immune cells promoting an antitumor response (**B**), and functional polarization of macrophages toward anti-inflammatory (M2) phenotype (**C**). Solid lines/arrows indicate activating or inhibiting effects on target immune cells. In correspondence of each line, the crucial molecular mediators carrying by TEVs are reported. Dotted arrows indicate the up or down-regulation of molecular targets in immune recipient cells.

The definition of the content of extracellular vesicles, and the understanding of how this often reflects that of the cell of origin, helps us to understand why cancer cells use the vesicles to alter the behavior of cells responsible for the immune surveillance. It is well known that vesicles can inhibit the immune response to cancer by acting directly on the components of the immune system, both at the innate and adaptive level [22]. Overall, TEVs act on the different immune cell types through three main mechanisms: functional activation, functional inhibition, and functional polarization (Figure 1). In particular, TEVs can inhibit the differentiation of myeloid and lymphoid progenitors as well as of dendritic cells (DCs), promote the expansion of myeloid-derived suppressor cells (MDSCs), inhibit the functions of natural killer (NK) cells, induce the apoptosis of CD8+ T cells, promote the expansion of Treg and Breg cells and foster the polarization of macrophages in M2 like-tumor associated macrophages (TAMs) [3]. We will, therefore, proceed to report and discuss the evidence of the effect of tumor-EVs in favoring the immunosuppression by analyzing separately the different cellular components involved (Figure 1).

### 2.1. Functional Activation

#### 2.1.1. TEVs and Myeloid-Derived Suppressor Cells

During tumor progression cancer cells can promote the expansion of myeloid-derived suppressor cells (MDSCs), a heterogeneous group of immature cells that derive from the myeloid lineage, with enhanced immunosuppressive activity within the tumor microenvironment [23]. By investigating the mechanisms by which tumor cells can contribute to MDSC expansion and activation, several groups focused on TEVs [24]. In particular, the ability of TEVs to promote MDSCs expansion has been attributed to their functional cargoes (Figure 1A). The first evidence was provided in 2009 [25]; in this study, authors have shown that murine breast carcinoma EVs are, in vivo, taken up by bone marrow precursor cells that in turn accumulated in the spleen and the tumor over 10 days. These cells showed MDSC functional characteristics and promoted tumor growth by inducing the release of the pro-inflammatory cytokine IL-6, Cox2, and VEGF. These effects were attributed to the presence of prostaglandin E2 (PGE2) and TGF-β in TEVs [25]. In addition to these EV-associated molecules, in another study, Hsp72 expressed at the surface of TEVs from three different tumor models (mammary, colon, and lymphoma cell lines), promoted MDSC suppressive activities. In particular, the authors showed that TEVs triggered in MDSCs the TLR2-mediated activation of the STAT3 pathway through the autocrine production of IL-6 [26]. Similarly, Multiple Myeloma-EVs enhance the immunosuppressive capacity of bone marrow MDSCs in mice, promoting their proliferation and survival by the activation of the STAT3 pathway [27]. More recently, the microRNA cargo of TEVs was deeply investigated and associated with the TEV-mediated MDSC expansion. Gastric cancer EVs delivered miR-107 to MDSCs, targeting DICER1 and PTEN, thus contributing to cell expansion and activation [28]. Further, the in vivo injection of miR-107 to mice promoted the accumulation of MDSC in the peripheral blood [28]. The MDSC-mediated immunosuppressive environment was largely described also in glioma [29]; recently, glioma-EVs were described as responsible for this event [30,31]. In a first study, authors showed that glioma EVs collected in hypoxia conditions had an increased ability to activate MDSCs compared to those collected in normoxia. This effect was correlated to the increased amount of EV- associated miR-10a and miR-21 that was able to activate MDSCs functions by targeting RAR-related orphan receptor alpha (RORA) and PTEN [30]. Further, the same group identified also TEV-associated miR-29a and miR-92a as responsible for MDSCs increased proliferation, but only miR-92a as the mediator of immunosuppressive functions [31].Further, data published in a very recent study, show that MDSCs, induced by glioblastoma-EVs, inhibit T cell proliferation [32].

#### 2.1.2. TEVs and Regulatory T cells

Regulatory T cells (Tregs) are a subpopulation of T cells, with immunosuppressive properties, also described as CD4+ FoxP3+. In physiologic conditions, they maintain self-tolerance by inhibiting T cell functions. Tregs are upregulated in cancer patients and contribute to creating an immunosuppressive microenvironment, favoring tumor progression [33,34,35]. Several studies were focused on correlating the increased amount of Tregs in cancer patients with TEV functions, analyzing if TEVs can directly affect this T cell population (Figure 1A). Szajnik and colleagues provided one of the first evidence in 2010; authors demonstrated that TEVs isolated from tumor cells induced the expansion of human Tregs, while EVs from normal cells did not [36]. In particular, after incubation with TEVs, Treg showed increased suppressor function by expressing FasL, IL-10, TGF-β1, CTLA-4, granzyme B, and perforin; cells were also resistant to apoptosis. Finally, the authors showed that TEV-associated TGF-β1 and IL-10 mediate Treg induction [36]. A further study from the same group showed that the increase of Treg suppression functions by TEVs did not depend on EVs uptake but is determined by receptor-ligand signaling on the cell surface [37]. Similarly, Wada and colleagues observed that malignant effusion-derived EVs contributed to the maintenance of Treg functions and the increased number of Treg cells in malignant effusions, by the membrane-associated TGF-β1 [38]. Also colorectal cancer cell-derived EVs were enriched with TGF-β1; TEVs were able to up-regulate Treg-related genes in Jurkat cells, PBMCs and T cells by activating Smad signaling [39]. TEVs from squamous cell carcinoma of the head and neck cell line, PCI-13, incubated with resting Tregs increased CD39 and CD73 expression as well as the production of adenosine, leading to the activation of Tregs. At the same time, in activated-Treg; incubation with TEVs leads to the up-regulation of immunosuppressive genes [40]. TEV-mediated Treg function increase was also described in nasopharyngeal carcinoma [41] and ovarian cancer [42].

### 2.2. Functional Inhibition

#### 2.2.1. TEVs and Dendritic Cells

Dendritic cells (DCs), also known as antigen-presenting cells (APCs), originated from the hematopoietic bone marrow progenitor cells and participate in the innate and adaptive immune response. In tumor microenvironment DCs act by capturing antigens and driving T cells to activate the antitumor immune response. The inhibition of DCs maturation is a mechanism through which tumors escape immune surveillance [43,44]. TEVs from murine mammary adenocarcinoma, human breast cancer, and melanoma cell lines interacted with bone marrow myeloid precursor cells and induce the production of IL-6 (Figure 1B); the cytokine release caused, partially, the block of the differentiation of myeloid precursor cells into DCs as shown by the low amount of the DC marker CD11c [45]. Recently, observations on EVs from Lewing lung carcinoma or breast cancer cells confirmed the TEV-mediated inhibition of myeloid precursor cell differentiation into DC; in this study authors also showed that EVs were able to induce DC apoptosis. Moreover, it was reported that TEV-treated DCs failed in inducing CD4+ T cell proliferation and activation, while promoted Treg differentiation. Finally, it has been shown that blockage of PD-L1 partially reversed the immunosuppressive effects induced by TEV–treated DCs [46]. In the lung cancer model, DCs become tolerogenic after incubation with EGFR-enriched TEVs; treated-DCs were then able to induce the generation of immunosuppressive Tregs [47].

#### 2.2.2. TEVs and Natural Killer Cells

Natural killer (NK) cells are a population of innate lymphocytes involved in the recognition of cancer cells; NK can cause the lysis or the apoptosis of the target cells without the involvement of other components and therefore, for this inherent capability, they are named “natural”. This population of cells has been largely described as “exhausted” in tumor since in this conditions NK cells exhibit a deregulated phenotype that consist in the reduction of cytokines production and expression of the NK-cell activating receptors and in the loss of lytic activity.

One of the first pieces of evidence on the role of TEVs on NK functions (Figure 1B) came in 2008 by Clayton and colleagues [48]. The authors isolated EVs from several in vitro cancer cell lines as well as from mesothelioma patients. After the addition of TEVs to NK and CD8+ T cells, the authors observed the downregulation of the NKG2D activating receptors. This effect was partially due to the presence of NKG2D ligands in TEVs but mainly to the presence of transforming growth factor (TGF)-β [48]. TGF- β is a crucial suppressive cytokine largely involved in NK cell exhaustion in cancer [49].

NK cell activity is inhibited in patients with acute myeloid leukemia (AML) [50]; this observation was correlated to the presence of transforming growth factor (TGF)-β in microvesiclesfrom the serum of AML patients [51]. Similar to the data reported in Clayton’s study, authors show that microvesicle-associated TGF-β down-regulated the expression of the NKG2D activating receptors leading to the impairment of NK cell functions [51].

The presence of TGF-β was also recently observed in EVs from highly metastatic pancreatic cancer cell lines and patient-derived primary cancer cells [52]. Authors show that pancreatic TEVs were able to downregulate the expression of NKG2D, CD107a, TNF-α, and INF-γ in NK cells leading to the impairment of NK cell functions. By a mass spectrometry approach, authors also showed that, in addition to TGF-β, TEVs are enriched in several immunosuppressive factors, such as nectin-2 and PVR that may explain the observed effects on NK cells. Wang et al. provided evidence on increased NK cell cytotoxicity by oral cancer-derived sEVs. The authors show that TEVs are enriched in the NF-κB-activating kinase-associated protein 1 (NAP1) that, in turn, activates the interferon regulatory factor 3 (IRF-3) pathway leading to cell cytotoxicity [53].

#### 2.2.3. TEVs and T lymphocyte Cells

T lymphocytes play a prominent role in the response to tumors; specifically in TME, by interacting with cancer cells and secreted factors, T cells became dysfunctional and exhibit decreased viability and proliferation and impaired effector functions [54]. In particular, CD8+ cytotoxic T lymphocytes are compromised in numerous cancer types [55] and several lines of evidence described EVs as mediators of the CD8+T cell growth inhibition (Figure 1B). EVs from head and neck squamous cell carcinoma and melanoma contained FasL and MHC class I molecules and significantly inhibit the expansion of primary-activated CD8+ T cells; consistent with these findings, authors showed that tumor EVs induce CD8+ Jurkat cells death [56]. Further studies by Liu and colleagues reported that also mice glioblastoma cells-EVs promote in vivo tumor cell growth by inhibiting CD8+T cell cytolytic activity [57]. In more recent studies, Maybruck and colleagues correlated the EV-mediated dysfunction of CD8+T cells, in head and neck cancer, with the presence of the immunoregulatory protein, galectin-1 [58], while in ovarian carcinoma, tumor EVs inhibit the proliferation of CD4+ and CD8+ T cells by delivering the metabolic checkpoint molecule arginase-1 [59]. One of the major mechanisms by which tumor cells can inhibit CD8+ T cell functions is correlated with activation of immune checkpoints as that mediated by the PD-1/PD-L1 pathway. As it is well known, tumor cells overexpress co-inhibitory receptor PD-L1 that binds the inhibitory molecule PD-1 on the activated T cells, driving to their inhibition [60,61]. Interestingly, accumulating evidence report that PD-L1 is contained and delivered by sEVs released many tumor types [62,63,64,65,66,67]; moreover, other studies revealed that TEVs can increase PD-L1 expression on monocytes [68,69]. The correlation between TEVs and PD-1/PD-L1 axis will be extensively discussed in paragraph 3.

### 2.3. Functional Polarization

#### TEVs and Macrophage Polarization

Tumor-associated macrophages (TAMs) represent an immune population of macrophages included in the tumor microenvironment. Their infiltration into the tumor correlates with an unfavorable prognosis, since they suppress the functions of cytotoxic T lymphocytes, thus supporting cancer progression and resistance to chemotherapy. TAMs have been shown to express an M2-like phenotype [70,71]. Recent studies showed that TEVs contributed to TAM polarization in particular in the acquisition of the M2 phenotype (Figure 1C). Colorectal cancer cell-derived EVs induced M2-like macrophage polarization by delivering miR-145, thus leading to the reduction of histone deacetylase 11. TEV-treated macrophages then promoted in vivo tumor growth [72].

Similarly, gastric cancer-derived EVs induced the expression of programmed cell death 1 (PD-1) in TAM, leading to IL10 production and CD8^+^T-cell function impairment [73]. We will extensively discuss how TEVs can modulate the PD1/PD-L1 axis in the last part of this review. Park and colleagues found that hypoxia contributes to the EV-mediated M2 polarization of TAM. Authors found that EVs released by hypoxic tumor cells were enriched in immunomodulatory proteins and chemokines including CSF-1, CCL2, FTH, FTL, and TGFβ that, together with the EVs-associated miRNAs, influence macrophage recruitment and promote M2-like polarization [74]. In addition to microRNAs, it was largely described that EVs carry functional long noncoding RNA (lncRNAs) [75] that contributed to the TEV-mediated tumor progression [76]. Recently, an interesting study by Liang and colleagues showed that lncRNA RPPH1 was up-regulated in colorectal cancer samples and associated with TEVs; also, authors found that the lncRNA can be transferred by EVs to macrophages, promoting their M2 polarization [77]. The studies mentioned and discussed above are summarized in Table 1.

## 3. Immune Checkpoints and Cancer

It is well-known that in physiological conditions, the immune effector cells drive potent anti-tumor responses, identifying and eliminating cancer cells with high specificity, based on the expression of “non-self” antigens [78,79,80]. However, “immunoparesis” or “lapse” of immune surveillance, which consequently leads to tolerance of growing cancer, is a common feature of the tumors [81,82,83,84,85,86,87,88,89]. The regulation of T cell response is the result of a balance between activating and repressing stimuli, also called immune checkpoints. Under physiologic conditions, T cells can recognize and specifically respond to foreign and native antigens to respectively eliminate pathogens and to maintain self-tolerance; two independent signaling pathways finely regulate this mechanism. T cell activation occurs through the formation of so-called “immunological synapse” [90]. The first signal of T cell activation during an immune response occurs through the binding of T cell receptor (TCR), on CD4+ helper T cells and CD8+ cytotoxic T cells, to the antigen which is held in the antigen-bearing major histocompatibility complex (MHC), on the surface of the APC (antigen-presenting cells). Additionally, T cells require secondary signals by co-stimulatory molecules that are critical for an effective immune response development [91,92]. The main determinant for co-stimulatory interaction is mediated by CD28-CD80 (B7-1)/CD86 (B7-2), respectively, on T cells and APC. In particular, CD28, expressed on T-cells, provides co-stimulatory signals required for T-cell activation and survival. Binding of B7-1/B7-2, on APCs, with CD28, on T-cells, enhances T-cell proliferation and IL-2 production. The absence of this simultaneous co-stimulatory signal results in T-cell dysfunction or anergy [93,94,95,96]. Apart from stimulatory signals that augment and sustain T-cell responses, inhibitory receptors, such as programmed death-1 (PD-1) or (CD279), CTLA-4, T-cell immunoglobulin, and mucin domain containing-3 (TIM-3) [97,98], lymphocyte-activation gene 3 (LAG-3) [99], or T-Cell immunoreceptor with Ig and ITIM domains (TIGIT) [100], are needed to down-regulate or inactivate T cells responses once the insult is eradicated. To limit the severity of the responses is necessary to avoid destructive action on healthy tissue and to maintain T cell’s intrinsic unresponsiveness to previously encountered antigens [101]. Cancer cells escape the control of the immune system [102] through several mechanisms; these include (i) the expression by tumor cells of “corrupted” versions of self molecules, (ii) the release of immunosuppressive substances and (iii) the aberrant expression of lymphocytes inhibitory receptor ligands. In addition, tumor cells can trigger an anti-tumor immune response by modulating macrophage functions [103]. Data in the literature showed that during the initial stages of tumorigenesis, immunosuppressive cytokines may promote anti-tumor responses; but, after long periods, they can promote cancer progression [104]. For example, TGF-β secretion inhibits the proliferation and effector functions of lymphocytes and macrophages. In addition to TGF-β, other immunosuppressive cytokines, such as vascular endothelial growth factor (VEGF), indolamine-pyrrole 2,3-dioxygenase (IDO) and IL10, act as immunosuppressive agents [105,106]. In other cases, macrophages with the M2 phenotype prevent the activation of T lymphocytes through the production of mediators such as IL-10 and prostaglandin E2 and promote tumor growth through the secretion of TGF- β and VEGF [107,108]. Alternatively, tumors may evade immune responses by taking advantage of PD-L1 macrophage expression that leads to T cell function suppression [109]. Cancer cells may further increase their resistance to cytotoxic T cell (CTL) attacks by increasing the expression of cell surface molecules such as Fas-ligand (Fas-L/APO-1-L/CD178). When bound to its respective surface receptor, Fas (CD95) expressed in T cells, FasL initiates pro-apoptotic signaling leading to apoptotic T cells death [110]. T cells express Fas as a safeguard to ensure that they can be destroyed to avoid potential autoimmune reactions; tumors, therefore, can exploit this mechanism to ensure their survival [111].

### 3.1. The Immune Checkpoint Inhibitor PD-1

Programmed death 1 receptor (PD-1; also called CD279) is an inhibitory receptor expressed on activated T cells, discovered in 1992 as a gene upregulated in murine T cell hybridoma undergoing cell death [112]. PD-1 (CD279) is expressed on CD4^−^CD8^−^thymocytes and CD4^+^CD8^+^ T cells during thymic development; furthermore, it is expressed on activated CD4^+^ and CD8^+^ T cells, on monocytes, natural killer T cells, B cells, and dendritic cells following activation mediated by certain“stimuli” [112,113].PD-1 receptor is a cell surface monomer consisting of a single immunoglobulin variable-like domain and a cytoplasmic domain containing two tyrosine-based signaling motifs: an immune receptor tyrosine-based inhibition motif, (ITIM), and an immune receptor tyrosine-based switch motif, (ITSM). The two PD-1 ligands PD-L1 (B7-H1; CD274) and PD-L2 (CD273) differ in their expression pattern. PD-L1 is constitutively expressed on T and B cells, on antigen-presenting cells such as dendritic cells (DCs), macrophages, monocytes, and mesenchymal stem cells. PD-L2 expression is much more restricted than PD-L1 being induced on DCs and macrophages [113]. Formation of PD-1/PD-L complex stimulates inhibitory intracellular signaling events due to the phosphorylation of tyrosine residues located in both ITIM and ITSM, and following recruitment of SH2 domain-containing tyrosine phosphatase 1 and 2 (SHP-1 and SHP-2); in a greater extent, SHP-2, induce de-phosphorylation and consequent inactivation of TCR signaling molecules as Zap70. This leads to the inhibition of T cell activity through reduction of proliferation, induction of apoptosis, and block of cytokine secretion such as IL-2, IFN-γ, and tumor necrosis factor (TNF)-α. [113,114,115]. Cancer cells can generate an immunosuppressive tumor microenvironment by expressing high amounts of inhibitory ligands such as PD-L1 and PD-L2, inhibiting the responses of T lymphocytes by binding to the PD-1 inhibitory receptor expressed by T lymphocytes, avoiding cytolysis by activated T cells towards cancer cells. PD-L1 expression was detected on many cancers including renal cell carcinoma [116], pancreatic cancer [117], breast cancer [118,119], gastric cancer [120,121,122], lung cancer [123,124], and colorectal cancer [125]. Ghebeb H et al. showed that PD-L1 expression in breast tumors was associated with high-risk clinicopathologic features, including high histologic grade and hormone receptor-negative status [118]. Mittendorf et al. identified PD-L1 expression in 20% of triple-negative breast cancer and suggest that PTEN loss is one mechanism regulating increased cell surface PD-L1 expression at the transcriptional level and that this effect occurs through the involvement of the phosphoinositide 3-kinase PI3K pathway activation. In particular, they demonstrated that activated T cells cultured with PTEN-silenced breast cancer cells showed decreased proliferation and increased apoptosis [119]. Beyond oncogenic signaling pathways such as PTEN, that can drive PD-L1 expression, other studies evaluated PD-L1 induction by several pro-inflammatory stimuli in cancer cells, particularly by interferons (IFNs), interleukin (IL)-6,IL-10, IL12, IL17, TGFβ, and TNFα, suggesting that multiple factors present in the tumor microenvironment may promote increased PDL1 expression by tumors [126]. Data in the literature showed that PD-L1 expression is higher in metastatic CRCs than in primary tumors [127]. Rosenbaum et al. demonstrated that PD-L1 in sporadic colorectal carcinomas is associated with signatures of colorectal carcinogenesis, including BRAF mutation, microsatellite instability, poor differentiation (with medullary morphology), and frequent tumor-infiltrating lymphocytes [125]. PD-L1 expression on tumor cells, detected both on the membrane and in the cytoplasm of tumor cells, is correlated with poor prognosis in NSCLC patients [124] and Azuma et al. [123] showed that expression of high-aggregated PD-L1 on tumor cells is associated with EGFR gene mutations. In gastric cancer patients, Hou et al. [122] showed a positive correlation between the expression of PD-L1 either on the membrane or in the cytoplasm of tumor cells and poor prognosis. Lu et al. identified a novel mechanism of PD-L1 activation in pancreatic ductal adenocarcinoma (PDAC); in particular, they showed that human mixed lineage leukemia protein-1 (MLL1) and PD-L1 are highly expressed in human PDAC specimens. MLL1, by binding to the H3K4 trimethylation (H3K4me3)—enriched promoter of the CD274 gene, promote the expression of PD-L1. Approaches aimed at decreasing PD-L1 expression, such as the MLL1 inhibitor Verticillin, were used in a preclinical model of pancreatic adenocarcinoma to improve the effects of anti-PD-l blockade antibodies [128]. Finally, PD-L1 is aberrantly expressed in clear cell renal cell carcinoma (ccRCC), the most common type of renal cell carcinoma (RCC) and this is often associated with increased risk of cancer mortality [116].

Programmed death 1 receptor (PD-1; also called CD279) is an inhibitory receptor expressed on activated T cells, discovered in 1992 as a gene upregulated in murine T cell hybridoma undergoing cell death [112]. PD-1 (CD279) is expressed on CD4^−^CD8^−^thymocytes and CD4^+^CD8^+^ T cells during thymic development; furthermore, it is expressed on activated CD4^+^ and CD8^+^ T cells, on monocytes, natural killer T cells, B cells, and dendritic cells following activation mediated by certain“stimuli” [112,113].PD-1 receptor is a cell surface monomer consisting of a single immunoglobulin variable-like domain and a cytoplasmic domain containing two tyrosine-based signaling motifs: an immune receptor tyrosine-based inhibition motif, (ITIM), and an immune receptor tyrosine-based switch motif, (ITSM). The two PD-1 ligands PD-L1 (B7-H1; CD274) and PD-L2 (CD273) differ in their expression pattern. PD-L1 is constitutively expressed on T and B cells, on antigen-presenting cells such as dendritic cells (DCs), macrophages, monocytes, and mesenchymal stem cells. PD-L2 expression is much more restricted than PD-L1 being induced on DCs and macrophages [113]. Formation of PD-1/PD-L complex stimulates inhibitory intracellular signaling events due to the phosphorylation of tyrosine residues located in both ITIM and ITSM, and following recruitment of SH2 domain-containing tyrosine phosphatase 1 and 2 (SHP-1 and SHP-2); in a greater extent, SHP-2, induce de-phosphorylation and consequent inactivation of TCR signaling molecules as Zap70. This leads to the inhibition of T cell activity through reduction of proliferation, induction of apoptosis, and block of cytokine secretion such as IL-2, IFN-γ, and tumor necrosis factor (TNF)-α. [113,114,115]. Cancer cells can generate an immunosuppressive tumor microenvironment by expressing high amounts of inhibitory ligands such as PD-L1 and PD-L2, inhibiting the responses of T lymphocytes by binding to the PD-1 inhibitory receptor expressed by T lymphocytes, avoiding cytolysis by activated T cells towards cancer cells. PD-L1 expression was detected on many cancers including renal cell carcinoma [116], pancreatic cancer [117], breast cancer [118,119], gastric cancer [120,121,122], lung cancer [123,124], and colorectal cancer [125]. Ghebeb H et al. showed that PD-L1 expression in breast tumors was associated with high-risk clinicopathologic features, including high histologic grade and hormone receptor-negative status [118]. Mittendorf et al. identified PD-L1 expression in 20% of triple-negative breast cancer and suggest that PTEN loss is one mechanism regulating increased cell surface PD-L1 expression at the transcriptional level and that this effect occurs through the involvement of the phosphoinositide 3-kinase PI3K pathway activation. In particular, they demonstrated that activated T cells cultured with PTEN-silenced breast cancer cells showed decreased proliferation and increased apoptosis [119]. Beyond oncogenic signaling pathways such as PTEN, that can drive PD-L1 expression, other studies evaluated PD-L1 induction by several pro-inflammatory stimuli in cancer cells, particularly by interferons (IFNs), interleukin (IL)-6,IL-10, IL12, IL17, TGFβ, and TNFα, suggesting that multiple factors present in the tumor microenvironment may promote increased PDL1 expression by tumors [126]. Data in the literature showed that PD-L1 expression is higher in metastatic CRCs than in primary tumors [127]. Rosenbaum et al. demonstrated that PD-L1 in sporadic colorectal carcinomas is associated with signatures of colorectal carcinogenesis, including BRAF mutation, microsatellite instability, poor differentiation (with medullary morphology), and frequent tumor-infiltrating lymphocytes [125]. PD-L1 expression on tumor cells, detected both on the membrane and in the cytoplasm of tumor cells, is correlated with poor prognosis in NSCLC patients [124] and Azuma et al. [123] showed that expression of high-aggregated PD-L1 on tumor cells is associated with EGFR gene mutations. In gastric cancer patients, Hou et al. [122] showed a positive correlation between the expression of PD-L1 either on the membrane or in the cytoplasm of tumor cells and poor prognosis. Lu et al. identified a novel mechanism of PD-L1 activation in pancreatic ductal adenocarcinoma (PDAC); in particular, they showed that human mixed lineage leukemia protein-1 (MLL1) and PD-L1 are highly expressed in human PDAC specimens. MLL1, by binding to the H3K4 trimethylation (H3K4me3)—enriched promoter of the CD274 gene, promote the expression of PD-L1. Approaches aimed at decreasing PD-L1 expression, such as the MLL1 inhibitor Verticillin, were used in a preclinical model of pancreatic adenocarcinoma to improve the effects of anti-PD-l blockade antibodies [128]. Finally, PD-L1 is aberrantly expressed in clear cell renal cell carcinoma (ccRCC), the most common type of renal cell carcinoma (RCC) and this is often associated with increased risk of cancer mortality [116].

### 3.2. TEVs as the Carriers of PD-L1

**Figure 2:**
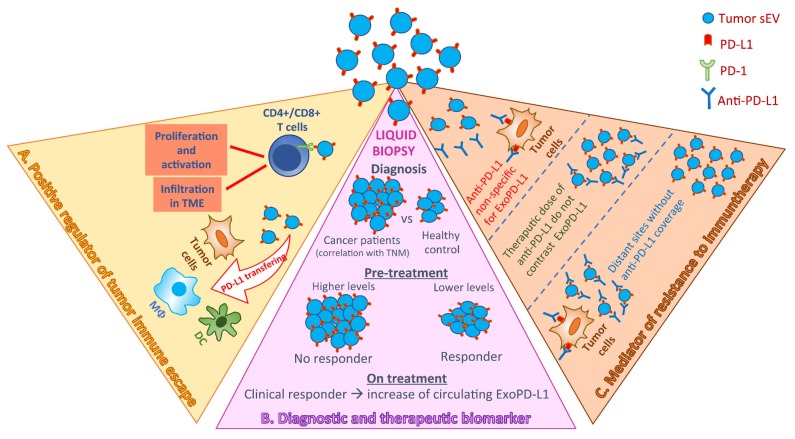
Summary of the biological and clinical meaning of tumor small extracellular vesicles (sEVs) carrying programmed death-1 (PD-1). Exosomal PD-L1 (ExoPD-L1) is described to play a direct role in altering immune surveillance (**A**), to have clinical relevance as a non-invasive tumor and immune cell biomarker in cancer (**B**), and it is considered as a potential mediator of resistance to current immunotherapeutic strategies (**C**).

The presence of PD-L1 in TEVs is largely reported in the literature (Figure 2). All available data have pointed out the direct role of exosomal PD-L1 (ExoPD-L1) in altering immune surveillance and its clinical relevance as a non-invasive tumor and immune cell biomarker in cancer [129,130,131].

Chen and colleagues have demonstrated that the sorting of PD-L1 in sEVs is not a random event, but it is specifically driven by ESCRT subunit HRS and Rab27a. Moreover, by using human melanoma xenografts in nude mice, they have shown that PD-L1 was present in circulating sEVs from mice bearing human melanoma xenografts but not from control mice [65]. Several studies have confirmed the presence of PD-L1^+^sEV in plasma and serum of patients with cancer [62,67,130,132]. An interesting study of Chulinget al. highlighted the clinical significance of circulating PD-L1 in head and neck squamous cell carcinoma (HNSCC). They found a significant difference in exosomal PD-L1 levels, but not in soluble PD-L1, between NSCLC patients and healthy controls. Moreover, the authors showed that levels of PD-L1 in sEV were correlated with TNM stage, tumor size, lymph node status, and distant metastasis, even if there was no correspondence with PD-L1 at the tissue level, revealed by immunohistochemistry (IHC) [62]. Diagnostic and prognostic value of circulating PD-L1^+^ sEVs was also reported in pancreatic ductal cancer [67].

Beyond the potential role as tumor biomarker, PD-L1 carried by TEVs is crucial in actively regulating tumor progression. The biological effects of ExoPD-L1 have been described in several in vitro and in vivo tumor models ([129] and references therein), such as murine and human HNSCC cell lines, melanoma, breast and prostate cancer, and glioblastoma. Data from all these studies showed that TEVs carrying PD-L1 have a critical role in inhibiting proliferation and activation of CD4+ and CD8+ T cells as well as their infiltration into the tumor microenvironment, drastically affecting the processes of immune surveillance and promoting tumor progression. Interestingly, it has also been reported that sEVs can transfer functional PD-L1 to multiple cell types including tumor cells, macrophages, and DCs, contributing to enhancing the immunosuppressive properties of the tumor microenvironment. Moreover, researchers have demonstrated that exosomal PD-L1 can bind to PD-1 thus directly inhibiting T-cell activities. These findings were further supported by in vivo data showing the ability of exosomal PD-L1 to promote tumor growth and, on the other side, the increase of activated T cells in mice injected with PD-L1^-^ sEVs. In the same study, it was also demonstrated that blockage of exosome-PD-L1 secretion inhibited the growth of breast cancer cells, and can increase the therapeutic efficacy of treatment with anti-PD-L1 [64]. Interestingly, glioblastoma-derived EVs drive the formation of non-classical monocytes (NCMs), CD14+/PD-1+/CD16+/HLA-DR^high^ [32], a specific immunosuppressive cell population widely described as anti-inflammatory [133,134]. In the same study, the authors reported that TEV-conditioned NCMs inhibit T cell proliferation and that the presence of PD-L1 in TEVs is responsible for this effect [32].

To date, the possibility to block the PD-1/PD-L1 pathway represents one of the most promising therapeutic approaches to contrast tumor progression. The use of immune checkpoints protein inhibitors, such as antibodies against PD-L1 (anti-PD-L1) and PD-1 (anti-PD-1), has shown notable long-term clinical benefits in a wide number of cancer types [135,136,137,138,139,140,141,142,143,144,145], even if the patient response rate is still low [146,147,148]. Thus, the understanding of mechanisms responsible for resistance to immunotherapy to predict patient response as well as the identification of biomarkers for proper patient selection is crucial for developing effective therapeutic strategies.

It has been widely reported that tumor PD-L1 is not a qualified biomarker to stratify patients for immunotherapy, since too often for patients having *PD*-L1 IHC staining positive anti-PD-1/PD-L1 therapy failed [146,149]. This discrepancy can occur due to temporal and spatial tumor heterogeneity and to the difficulty of performing biopsies at multiple time points. In this perspective, the use of liquid biopsy is strongly encouraged since it can be easily repeated over time and can reflect the overall state of a tumor, overcoming the problems associated with heterogeneity [150].

Interestingly, the numerous studies exploring the clinical significance of ExoPD-L1 highlighted that, in addition to be correlated with tumor growth and progression, ExoPD-L1 is responsible for mediating resistance to immunotherapy and it should be considered as a potential biomarker in predicting the outcome of anti-PD-1/PD-L1 blockade therapy. Even if the mechanism by which ExoPD-L1 can cause resistance is still unclear, several possible scenarios have been depicted. It was proposed that the exposure of PD-L1 on sEV surface makes it less responsive to used antibodies or that the therapeutic dose of anti-PD-L1 is not enough to contrast the high levels of exosomal PD-L1. Finally, sEVs can carry PD-L1 on targets out of control of antibody [151]. Anyway, it is clear that a deeper comprehension of the mechanisms mediated by ExoPD-L1 could be attractive for improving the current therapeutic strategies.

The role of ExoPD-L1 as biomarkers of response to oncological immunotherapy was reported for metastatic melanoma. Chen and colleagues showed that pre-treatment and on-treatment level of circulating ExoPD-L1 was correlated with clinical outcome for patients treated with pembrolizumab reflecting distinct states of anti-tumor immunity. They found higher pre-treatment levels of exosomal PD-L1 in non-responder patients and during pembrolizumab therapy observed a progressive significant increase of PD-L1 on circulating sEVs for the clinical responders [65].

Collectively, data here discussed suggest that PD-L1 carried by sEVs actively contributes to attenuate anti-tumor immunity, is the mediator of immunotherapy resistance and can be considered a good diagnostic and therapeutic biomarker. Further characterization of biological activities and clinical meaning of ExoPD-L1 will contribute to better comprehend the mechanisms of tumor immune escape and to improve the efficacy of therapeutic strategies.

### 3.3. TEVs as Modulators of PD-L1 Expression in Target Cells

Recent evidence suggests that sEVs released by cancer cells not only carry PD-L1 on their surface but are also able to induce its expression in myeloid cells (Figure 1C), thus further contributing to enhancing the immunosuppressive status of the tumor microenvironment (TME). Haderkand colleagues showed that sEVs derived from chronic lymphocytic leukemia (CLL) cells induced monocytes to acquire a skewed pro-tumorigenic phenotype characterized by the increase of cytokine release and PD-L1 expression. The authors identified lncRNA as the molecular mediator of CLL- sEVs effects; in fact, the same result was obtained by treating monocytes with the exogenous noncoding Y RNA hY4, found significantly enriched in both CLL-derived sEVs and sEVs from the plasma of CLL patients [152]. Moreover, they propose that hY4-induced responses were dependent on endosomal TLR7 and consistent with this hypothesis, they found that upon treatment with CLL-derived sEVs or hY4 the pharmacological inactivation of TRL7 significantly reduced cytokine release and PD-L1 expression in monocytes in vitro and attenuated CLL development in vivo. These observations offer great potential fordeveloping new combinatorial therapeutic strategies which include exosomal targets [152]. Glioblastoma (GBM)-derived sEVs have been also found to elicit in monocytes the PD-L1 upregulation and the skewing towards immune suppressive M2 phenotype. Interestingly, these effects were observed in PD-L1 CD14+ monocyte/macrophages isolated from GBM tissue but not in those obtained from the blood of GBM patients and healthy donors. Finally, it was reported that the action of sEVs was partly mediated by activation of STAT3 pathways as indicated by the increasing of pSTAT3 levels [69].

Similar results were obtained by using a mouse melanoma in vitro model where a different molecular mechanism underlying the immunosuppressive potential of TEVs was described. Indeed, in this study, it was reported that the ability of melanoma cell-derived EVs to induce PD-L1 expression on immature myeloid cells was dependent on the expression on target cells of Toll-like receptors (TLR) activated by binding of HSP86 carried by EVs [68].

Within the tumor microenvironment, not only TEVs are able to affect the behavior and properties of surrounding immune cells. It was demonstrated that sEVs derived from breast cancer-derived primary mesenchymal stem cells (MSCs) induce differentiation of monocytic myeloid-derived suppressor cells (M-MDSCs) into M2-polarized macrophages and the upregulation of PD-L1 expression. This interesting study highlight as the crosstalk between tumor, mesenchymal and immune cells mediated by sEVs drives breast cancer progression by prompting myeloid cells to become immunosuppressive macrophages, thus establishing a crucial requirement for promoting tumor growth [153]. These findings highlight novel EV-mediated mechanisms inducing myeloid cells into skewed immunosuppressive phenotype thus supporting tumor growth.

## 4. Conclusions

In conclusion, here we reported the evidence of the involvement of TEVs as contributors to the cancer-immune escape, highlighting their role in modulating the PD-L1/PD-1 axis. The studies discussed above suggest thatthe development of EV targeting strategies can improve anti-cancer immunotherapies. Some approaches are conceivable and part of the scientific community in the field is focused on these; for example, inhibiting the release of vesicles by cancer cells, or blocking their specific interaction with target cells isunder investigation. However, although promising, the detailedexamination of vesicle content, combined with the in-depth comprehension of the in vivo mechanisms underlying TEV-mediated immune escape, are necessary steps for the furtherclinical application.

## Figures and Tables

**Table 1:** Summarizing the evidence for the role of tumor-derived extracellular vesicles (TEVs) in tumor-mediated immune suppression.

Tumor Models	TEV Cargo	Target Cells	Biological Effects	References
**Functional Activation**				
Breast cancer	PGE2 and TGF-β	MDSCs	Tumor growth promotion by inducing the release of IL-6, Cox2, and VEGF	[25]
Breast, colon cancer and lymphoma	Hsp72	MDSCs	Immunosuppressive capacity promotion by activating STAT3 pathway	[26]
Gastric cancer	miR-107	MDSCs	Cell expansion and activation by targeting DICER1 and PTEN	[28]
Glioma	miR-10a and miR-21	MDSCs	Cell functions activation by targeting RAR-related orphan receptor alpha (RORA) and PTEN	[30]
Glioma	miR-29a and miR-92a	MDSCs	Increased proliferation and immunosoppressive function promotion	[31]
Ovarian cancer	TGF-β1 and IL-10	Treg	Increased immunosuppressive capacity by expressing IL-10, TGF-β1, CTLA-4, granzyme B	[36]
Colorectal cancer	TGF-β1	Treg	Up-regulation of Treg-related genes by activating SMAD pathway	[39]
**Functional Inhibition**				
Lung cancer	EGFR	DCs	Tolerogenic induction	[47]
Mesothelioma	NKG2D ligands and TGF-β	NK and CD8+	Cell exhaustion through NKG2D activating receptors down-regulation	[48]
Pancreatic cancer	TGF-β, nectin-2 and PVR	NK	Cell functions impairment by down-regulating NKG2D, CD107a, TNF-α, and INF-γ expression	[52]
Oral cancer	NAP-1	NK	Increased cell toxicity by activating the interferon regulatory factor 3 (IRF-3) pathway	[53]
Head and neck cancer, melanoma	FasL and MHC class I molecules	CD8+	Cell expansion inhibition and cell death induction	[56]
Head and neck cancer	Galectin-1	CD8+	Cell dysfunction induction	[58]
Ovarian carcinoma	Arginase I	CD4+ and CD8+	Cell proliferation inhibition	[59]
Gliobastoma	PD-L1	CD4+ and CD8+	Cell activation and proliferation inhibition	[63]
Breast cancer	PD-L1	CD4+ and CD8+	Cell activation inhibition	[64]
**Functional Polarization**				
Colorectal cancer	miR-145	macrophages	Tumor growth promotion through M2-like macrophage polarization induction	[72]
Melanoma, skin, lung cancer	CSF-1, CCL2, FTH, FTL, TGFβ	macrophages	Tumor growth promotion through M2-like macrophage polarization induction	[74]
Colorectal cancer	lncRNA RPPH1	macrophages	Tumor growth promotion through M2-like macrophage polarization induction	[77]
Glioblastoma	STAT3 pathway activator	monocytes	Tumor growth promotion through M2-like macrophage polarization induction	[69]

## Abbreviations

EVs	Extracellular vesicles
TEVs	Tumor-derived extracellular vesicles
sEVs	Small extracellular vesicles
TME	Tumor microenvironment
Tregs	Regulatory T cells
MDSCs	Myeloid-derived suppressor cells
mRNAs	Messenger RNAs
miRNAs	microRNAs
lncRNAs	long non-coding RNAs
DCs	Dendritic cells
NK	Natural killer
APCs	Antigen-presenting cells
TAMs	Tumor-associated macrophages
CTLs	cytotoxic T lymphocytes
PD-1	Programmed cell death 1
PD-L1	Programmed death-ligand 1
